# Rapid diagnosis of acanthamoeba keratitis using non-nutrient agar with a lawn of *E. coli*

**DOI:** 10.1186/1869-5760-3-40

**Published:** 2013-02-27

**Authors:** Samuel Borin, Ilan Feldman, Shifra Ken-Dror, Daniel Briscoe

**Affiliations:** 1 St Bartholomew's and the Royal London School of Medicine, Whitechapel Road, London, E1 1BB, UK; 2 Department of Ophthalmology, Emek Medical Centre, Afula, 18101, Israel; 3 Clinical Microbiology Laboratory, Haifa and W. Galilee District Laboratories, Haifa, 34992, Israel

**Keywords:** Acanthamoeba, Keratitis, Corneal ulcer, Culture, Orthokeratology, Cornea

## Abstract

**Background:**

A patient presented with a corneal foreign body in his only eye. He was treated with prophylactic antibiotics and sent home, but deteriorated.

**Findings:**

He returned to the hospital 5 days later, and on slit-lamp examination, there was ciliary injection, corneal oedema and a 1 mm × 1 mm corneal abscess with mild anterior uveitis. Corneal scrapings were taken for culture on a non-nutrient agar with a lawn of *Escherichia coli*, on chocolate agar and on blood agar. He was treated with fortified gentamicin and cefazolin drops. He improved and was discharged 4 days after admission. On day 5, the culture results showed acanthamoeba. He was brought back to the hospital and treated with hourly chlorhexidine drops, ofloxacin six times daily and neomycin/dexamethasone drops once daily. On day 7, he was discharged to continue treatment at home, at which time his visual acuity in that eye was 6/9, and slit-lamp examination showed punctate keratitis and a stromal opacity with mild peripheral infiltration.

**Conclusions:**

Culture on non-nutrient agar with a lawn of *E. coli* is a rapid, reliable and less invasive alternative to corneal biopsy for the diagnosis of acanthamoeba infection. We suggest using this method where acanthamoeba is suspected. Owing to the risk of corneal abscess, orthokeratology should be avoided in an amblyopic patient or an only eye. Acanthamoeba infection may be masked by other eye diseases.

## Findings

### Introduction

Acanthamoeba is a ubiquitous protozoan and a rare causative organism for keratitis, representing 0.15 per million cases of keratitis in the USA; 70% to 85% of cases of acanthamoeba keratitis are associated with contact lens use [1]. In acanthamoeba keratitis, re-epithelialisation takes longer than that in keratitis caused by other organisms, and the visual outcome is generally worse. The disease may be diagnosed by corneal biopsy and by culture on non-nutrient agar with a lawn of *Escherichia coli*. There is often a relatively long period of milder symptoms before the infection becomes severe. It often responds poorly to first-line antimicrobial treatments.

Orthokeratology is a controversial technique for correcting myopia. It has been in use since the 1960s. It uses contact lenses which are worn to flatten the cornea, reducing its refractive power. These lenses were originally made from polymethyl methacrylate, but are now made of rigid gas-permeable materials. Once the cornea reaches the desired shape, maintenance lenses are used overnight. The user can then see during the day without the need for corrective glasses or contact lenses. There are significant complications associated with orthokeratology, including corneal abscess, corneal epithelial oedema, corneal abrasions or staining, keratoconus, corneal thinning, corneal scarring, corneal warpage and induced astigmatism [2]. Damage to the corneal epithelium increases the risk of corneal infection. This damage is likely to be greatest at the central cornea, due to the relative curvature of the cornea and the lenses. Therefore, infections often occur centrally and have a severe effect on the patient's vision. The use of overnight lenses is also a risk factor for corneal infection [3]. This may be because of the higher temperature under the closed lid and the absence of blinking and tearing at night [4]. Due to the high risk of severe complications, the use of these lenses is very controversial.

We report a case of acanthamoeba keratitis in an amblyopic orthokeratology patient which was rapidly diagnosed using the *E. coli* lawn method.

### Case

In 2011, a patient presented to the hospital with a corneal foreign body in his left good eye. He suffered from deep amblyopia and strabismus in his right eye, myopia and astigmatism. Of note in his history were the use of orthokeratology lenses and a deep amblyopic right eye due to strabismus/unilateral myopia in childhood. The foreign body was removed, prophylactic antibiotic drops were given and the patient was advised to refrain from using his orthokeratology lenses. He re-presented 5 days later complaining of a painful, red left eye with secretions. On examination, the left eyelid was swollen. His visual acuity in the left eye was 6/20, and on slit-lamp examination, there was ciliary injection, corneal oedema, a 1 mm × 1 mm corneal abscess and a ring infiltrate typical of acanthamoeba with mild anterior uveitis. His pain was not as severe as is typical in such cases. The examination was otherwise normal. The patient was admitted to the hospital, cultures were taken from corneal scrapings and treatment was started with fortified gentamicin and cefazolin drops. Over the next 4 days, his cornea improved and he was discharged. On day 5 after admission, the culture results showed acanthamoeba. These cultures were done on a non-nutrient agar with a lawn of *E. coli*, a technique which allows rapid diagnosis without the need for a corneal biopsy. Fungal and bacterial cultures were negative. He was readmitted to the hospital despite his improvement, and treatment was changed to hourly chlorhexidine drops, ofloxacin six times daily and neomycin/dexamethasone drops once daily. He was discharged after 7 days to continue treatment at home, at which time his visual acuity in the left eye was 6/9, and slit-lamp examination showed punctate keratitis and a stromal opacity with mild peripheral infiltration.

#### Questions

The following are the questions raised by this case:

1. What method is best for the diagnosis of acanthamoeba keratitis?

2. Should this patient have been using orthokeratology?

3. What could be done to improve the outcome in similar cases?

#### Answers

The following are our answers:

1. What method is best for the diagnosis of acanthamoeba keratitis?

The laboratory at Emek Medical Centre uses a non-nutrient agar with a lawn of *E. coli* for culturing acanthamoeba. This allows diagnosis without a corneal biopsy, which should be avoided if possible. We suggest that such a culture is a superior method for diagnosing acanthamoeba and should be adopted wherever it is possible.

2. Should this patient have been using orthokeratology?

This amblyopic patient was using orthokeratology lenses in his good eye, and this certainly contributed to the acanthamoeba infection. In our opinion, the use of orthokeratology lenses in an amblyopic patient or in an only eye should be avoided.

3. What could be done to improve the outcome in similar cases?

Diagnosis of acanthamoeba may be delayed because other diseases may mask it or other organisms may seem to be a more likely cause of the patient's symptoms. Delayed diagnosis is also associated with worse visual outcomes, making prompt diagnosis especially important. A culture on non-nutrient agar with a lawn of *E. coli* should always be done to look for acanthamoeba in corneal abscess associated with contact lenses, given the poor outcome and insidious onset of this disease.

## Consent

Written informed consent was obtained from the patient for publication of this report.

## Competing interests

The authors declare that they have no competing interests.

## Authors' contributions

SB and IF contributed to the acquisition, analysis and interpretation of data; drafted the article; revised it critically for important intellectual content and approved the version to be published. SK-D contributed to the acquisition of data, revised the article critically for important intellectual content and approved the version to be published. DB contributed to the acquisition, analysis and interpretation of data; revised the article critically for important intellectual content and approved the version to be published. All authors read and approved the final manuscript.
